# Unilateral subcutaneous fibroma in the distal femoral region of a 5-year-old Nooitgedacht mare

**DOI:** 10.4102/jsava.v89i0.1636

**Published:** 2018-12-05

**Authors:** Luke A. Poore, Neil Duncan, June Williams

**Affiliations:** 1 Department of Companion Animal Clinical Studies, University of Pretoria, South Africa; 2 Department of Paraclinical Studies, University of Pretoria, South Africa

## Abstract

A non-ossified unilateral subcutaneous fibroma was diagnosed in the distal femoral region of a 5-year-old Nooitgedacht mare. Histopathological examination of the excised mass revealed long interweaving bundles of semi-mature monotonous collagenous connective tissue with fusiform nuclei without mitotic figures. The mare made an uneventful recovery following surgical removal of the neoplasm. Subcutaneous fibromas should be considered in the differential diagnosis of skin swellings associated with the limbs of horses.

## Introduction

Neoplasia of the appendicular skeleton in the horse is unusual (Schooley & Hendrickson 1998), with the axial skeleton and head being more commonly affected (Collins 1998; Gibbs 1994; Hance & Bertone 1993; Kidd & Bradshaw 2002). Cutaneous fibromas have rarely been reported in domestic animals, although they are common in humans (Jacobson 1971). By contrast, the tumour often confused with fibroma, and often called fibroma, is bovine papilloma virus–associated equine sarcoid, which is the most common tumour of horses and accounts for over half of all equine skin tumours (Taylor & Haldorson 2013). There are a small number of previous reports of non-sarcoid cutaneous or subcutaneous fibroma in the horse (Attenburrow & Heyse-Moore 1982; Kidd & Bradshaw 2002). This, to the authors’ knowledge, is the first report of a confirmed non-ossifying subcutaneous fibroma on the limb of a horse.

## Ethical considerations

No approval was required as this case report describes a clinical case and the mare was part of the teaching herd of the Onderstepoort Veterinary Academic Hospital, University of Pretoria.

## Case presentation

A 5-year-old Nooitgedacht mare was presented to the Onderstepoort Veterinary Academic Hospital, University of Pretoria, South Africa for evaluation of a mass overlying the right distolateral femoral region. It was reported that the mass had been present for approximately 3 months and had slowly increased in size during this period. In addition, the mare had shown evidence of right hind limb lameness of increasing severity over this period. Clinical examination revealed a firm, single smooth nodular subcutaneous mass measuring 4 cm × 5 cm below but adherent to the skin at the distolateral aspect of the right femur. The mare resented local palpation. No other abnormalities were evident on a general physical examination. A dynamic gait evaluation performed in hand at a trot on a straight line on a hard surface revealed a 3/5 right hind limb lameness (American Association of Equine Practitioners Scale 1–5).

A radiographic examination of the distal right femur was unremarkable and ultrasonographic evaluation revealed a well-defined subcutaneous homogenous mass with moderate echodensity. No abnormalities were palpable or visualised ultrasonographically at the local or regional lymph nodes to indicate metastasis.

**FIGURE 1:**
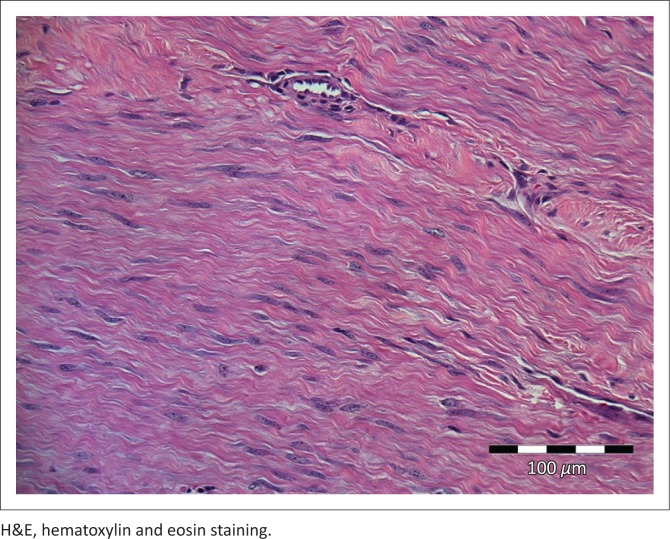
A photomicrograph showing the fibrous connective tissue of the mass in longitudinal section with elongated wavy collagen fibrils and nuclei. (H&E ×400).

**FIGURE 2:**
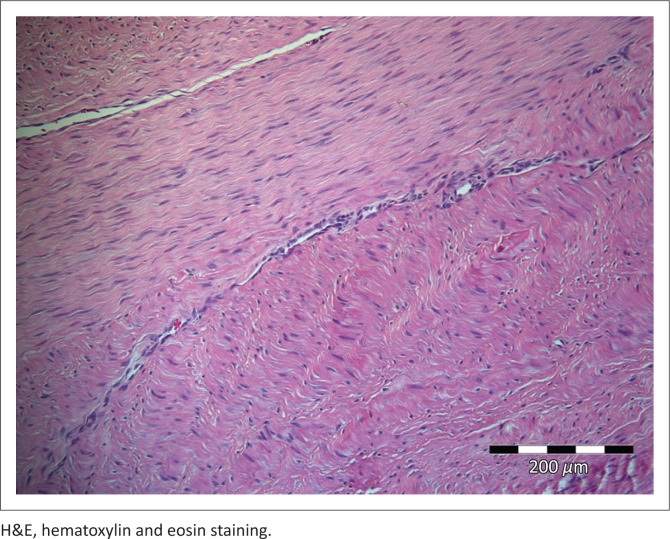
A photomicrograph showing the fibroma exhibiting interweaving regions of fairly well-differentiated but monotonous collagenous tissue. (H&E ×200).

The mass, which was of firm consistency and grey to white on cut surface as previously reported (Hendrick 2002), was surgically excised. Histopathological examination, using routine haematoxylin and eosin staining on sections cut from formalin-fixed, paraffin wax-embedded tissue, revealed long interweaving bundles of semi-mature monotonous collagenous connective tissue. Mitotic figures were absent and the nuclei were fusiform and regular to wavy. Occasional blood vessels showed a mild perivascular round cell infiltrate. A diagnosis of fibroma was made (Figures 1 and 2).

Immunohistochemical examination was performed by the avidin–biotin complex technique (Haines & Chelack 1991) using Polyclonal Rabbit Anti-S100 (Dako Code No. Z0311; Dako, California, United Stated) at the dilution of 1:32 000, after antigen retrieval at pH6, antibody incubation time of 1 hour, and using the Dako Real En Vision Detection System (Ref K5007), to exclude spindle cells of neural origin. The result was diffusely negative when compared with the labelling of a section of spinal cord in which glial cells labelled correctly. This was specifically done to exclude the differential diagnosis of cutaneous schwannoma, which is a benign spindle cell variant of the four subtypes of peripheral nerve sheath tumours, and occasionally seen in the dermis of horses (Higgins et al. 2017; Schöniger et al. 2011).

The mare was re-examined as an outpatient 14 days later when skin staples and sutures were removed. She was clinically unremarkable, the swelling of the surgical site had resolved and the area was non-painful on palpation. The mare was sound and comfortable when trotted on a straight line on a hard surface. She was maintained in a small paddock and the surgical site monitored for swelling or discharge for 6 weeks. At examination 12 months post-surgery the mare was clinically healthy, the surgical site had healed without tumour recurrence and she remained sound.

## Discussion

Fibromas are benign neoplasms composed of fibrous tissue or fully developed connective tissue (Kidd & Bradshaw 2002). Fibromas account for between 2.1% and 17.1% of all equine skin neoplasms (Scott 2007). There has been significant controversy differentiating fibromas from equine sarcoids (Baker & Leyland 1975; Valentine 2006) and no breed or sex predilection has been reported (Baker & Leyland 1975; De Meyer et al. 2017).

Fibromas have been predominantly reported to be associated with the equine head with affected regions, including the guttural pouch (Merriam 1972), orbit (Colitz et al. 2000), mandible (Morse et al. 1988; Robbins, Arighi & Ottewell 1996; Sponseller et al. 2006), maxilla (Kodaira et al. 2010), nasal cavity (Puff et al. 2006) and paranasal sinuses (Cilliers et al. 2008; Dixon & Head 1999). These have frequently been ossifying fibromas (Cilliers et al. 2008; Kodaira et al. 2010; Morse et al. 1988; Puff et al. 2006; Robbins et al. 1996; Sponseller et al. 2006).

There are two reports of fibroma associated with the equine abdomen (Tafti, Oryna & Maleki 1998; Wilson & Sykes 1981), one with the neck (McCauley et al. 2002) and one with the prepuce (De Meyer et al. 2017).

Fibromas associated with the appendicular skeleton have been reported infrequently. These include ossifying fibroma of the proximal tibia (Collins 1998), non-ossifying fibroma of the proximal tibia (Kidd & Bradshaw 2002), fibroma of the tendons (Adams, Fessler & Thacker 1982) and non-ossifying fibroma of the proximal phalanx (Attenburrow & Heyse-Moore 1982). No reports were found of cutaneous or subcutaneous fibroma associated with the limbs.

Fibromas in large animals have been described as solitary, slow growing, well-defined, progressive, round to oval intradermal or subcutaneous masses (Scott 1988). They are usually firm, white to tan on cut surface and well demarcated but not encapsulated in horses (Knottenbelt, Patterson Kane & Snalune 2015). Although classified as benign lesions, surgical resection has been recommended and recurrence has been reported with incomplete excision (Colitz et al. 2000). With complete resection, recurrence is rare (Knottenbelt et al. 2015).

Palpation, radiographic examination and ultrasonographic examination of the mass in this case report showed it to be well defined in the subcutaneous tissue overlying the right lateral distal femoral region. The overlying epidermis was intact, was dissected away from the mass and there was no evidence of alopecia, hypopigmentation or ulceration, which have been reportedly associated with fibromas (Scott 2007; Scott & Miller 2003).

Histologically, fibroma is characterised by a low number of fibroblasts with interwoven collagen fibres (De Meyer et al. 2017). These fibroblasts are fusiform with a low mitotic index (De Meyer et al. 2017). Collagenous fibres are variable in maturation and have a haphazard orientation (De Meyer et al. 2017).

Essential characteristics of equine sarcoid that differentiate it from fibroma include the hyperplastic, hyperkeratotic epithelial component with typical long, slender extensions (rete pegs) into the proliferating immature dermal fibroblasts (Martens et al. 2000; Taylor & Haldorson 2013), which show mitotic figures in a roughly whorled fibrocellular mass (Taylor & Haldorson 2013). These were not evident in the current case. Fibromas do not usually have an epithelial component, whereas, below the epithelium in sarcoids, except in nodular or early occult varieties (Mauldin & Peters-Kennedy 2017), the superficial fibroblasts classically are orientated perpendicularly to the basement membrane (Bogaert et al. 2008; Bogaert et al. 2010; Martens et al. 2000). Six variants of sarcoid are described, and they are commonly found in various sites on the head, neck, extremities and the ventrum, including the groin. They may occur singly or multifocally (Mauldin & Peters-Kennedy 2017). The epidemiology of sarcoid, in contrast to fibroma, is also specific and unique, being associated with bovine papillomavirus (BPV-1, BPV-2 and BPV-13) (Mauldin & Peters-Kennedy 2017), with potential risks being genetic haplotype of the horse, the presence of fly vectors and skin trauma (Taylor & Haldoson 2013). For this reason, the difference between sarcoid and fibroma can be made based on PCR for BPV.

Schwannomas, a recently recognised and reported differential diagnosis for fibroma and sarcoid, are now classified as a subtype of peripheral nerve sheath tumours (PNST) (Higgins et al. 2017). They are uncommonly recognised in horses, and are solitary, well-demarcated, expansile, usually dermal, spindle cell masses which may be multinodular or single; all 22 cases in one series labelled positively with anti-S100 antibody (Schöniger et al. 2011). Occasional cutaneous spindle cell tumours in horses that appear histologically to overlap between sarcoid and PNST may variably label with S100 antibody (Drs J. Steyl and S. Clift, Onderstepoort, South Africa, pers. comm., 04 January 2018). The fibroma in the current case was clearly immunohistochemically negative for S100.

Various options have been proposed for the treatment of fibroma in the horse with surgical curettage (Attenburrow & Heyse-Moore 1982), radiotherapy (Orsini, Baird & Ruggles 2004; Robbins et al. 1996; Wyn-Jones 1983), carbon dioxide laser (McCauley et al. 2002) and cisplatin-containing biodegradable beads (Hewes & Sullins 2006) suggested.

To our knowledge, there are no previous reports of limb-associated subcutaneous fibromas (non-ossifying) in the horse. Subcutaneous fibromas should be considered in the differential diagnosis of skin swellings associated with the limbs of horses. Surgical excision of the mass in this case report resulted in a full resolution of the clinical signs and is recommended in the treatment of this form of benign neoplastic mass in horses.
